# Observed efficacy and clinically important improvements in participants with osteoarthritis treated with subcutaneous tanezumab: results from a 56-week randomized NSAID-controlled study

**DOI:** 10.1186/s13075-022-02759-0

**Published:** 2022-03-29

**Authors:** Tuhina Neogi, David J. Hunter, Melvin Churchill, Ivan Shirinsky, Alexander White, Ali Guermazi, Masanari Omata, Robert J. Fountaine, Glenn Pixton, Lars Viktrup, Mark T. Brown, Christine R. West, Kenneth M. Verburg

**Affiliations:** 1 Boston University School of Medicine, Boston, MA USA; 2 University of Sydney, Sydney, Australia; 3 Arthritis Center of Nebraska, Lincoln, NE USA; 4 Federal State Budgetary Scientific Institution Research Institute of Fundamental and Clinical Immunology, Novosibirsk, Russia; 5 Progressive Medical Research, Florida, FL USA; 6 Ohimachi Orthopaedic Clinic, Tokyo, Japan; 7 Pfizer Inc., Groton, CT USA; 8 Pfizer Inc., Morrisville, NC USA; 9 Eli Lilly & Company, Indianapolis, IN USA

**Keywords:** Tanezumab, Nonsteroidal anti-inflammatory drugs, Osteoarthritis, Nerve growth factor, Clinical-trial, Efficacy

## Abstract

**Background:**

A recent phase 3 study demonstrated that treatment with tanezumab, a nerve growth factor inhibitor, or nonsteroidal anti-inflammatory drugs (NSAIDs) improves pain and physical function in participants with moderate-to-severe osteoarthritis (OA) of the hip or knee. Here, we evaluated the time course and clinical importance of these initial efficacy findings using a mixture of primary, secondary, and post hoc endpoints.

**Methods:**

Participants on stable NSAID therapy and with a history of inadequate response to other standard OA analgesics were enrolled in an 80-week (56-week treatment/24-week safety follow-up), randomized, NSAID-controlled, phase 3 study primarily designed to assess the safety of tanezumab for moderate-to-severe OA of the knee or hip. Participants received oral NSAID (twice daily naproxen, celecoxib, or diclofenac) or subcutaneous tanezumab (2.5mg or 5mg every 8 weeks). Non-responders were discontinued at week 16. Changes from baseline in WOMAC Pain and Physical Function, Patient’s Global Assessment of Osteoarthritis (PGA-OA), and average pain in the index joint were compared between tanezumab and NSAID groups over the 56-week treatment period. Clinically meaningful response (e.g., ≥30% and ≥50% improvement in WOMAC Pain and Physical Function), rescue medication use, and safety were also assessed.

**Results:**

All groups improved WOMAC Pain, WOMAC Physical Function, PGA-OA, and average pain in the index joint over the 56-week treatment period relative to baseline. Across all groups, improvements generally occurred from the time of first assessment (week 1 or 2) to week 16 and then slightly decreased from week 16 to 24 before stabilizing from weeks 24 to 56. The magnitude of improvement and the proportion of participants achieving ≥30% and ≥50% improvement in these measures was greater (unadjusted *p*≤0.05) with tanezumab than with NSAID at some timepoints on or before week 16. Adverse events of abnormal peripheral sensation, prespecified joint safety events, and total joint replacement surgery occurred more frequently with tanezumab than with NSAID.

**Conclusions:**

Tanezumab and NSAID both provided early and sustained (up to 56 weeks) efficacy relative to baseline. Improvements in pain and function were clinically meaningful in a substantial proportion of participants. Adverse events of abnormal peripheral sensation and joint safety events occurred more frequently with tanezumab than with NSAID.

**Trial registration:**

ClinicalTrials.govNCT02528188. Registered on 19 July 2015.

**Supplementary Information:**

The online version contains supplementary material available at 10.1186/s13075-022-02759-0.

## Background

Osteoarthritis (OA) is the most common form of arthritis and represents a significant global burden, yet few safe and effective therapies exist [1]. A combination of pharmacological, physical, and behavioral approaches is recommended for the management of OA, though recent guidelines highlight the need for new treatment options [2, 3]. The cornerstone of pharmacologic treatment is nonsteroidal anti-inflammatory drugs (NSAIDs), which demonstrate efficacy for OA-related pain in short-term clinical trials and are typically recommended over other pharmacological agents [2, 4, 5]. However, due to the potential for gastrointestinal, cardiovascular, and renal complications, their use is not recommended in patients at risk for these adverse events (AEs) and their long-term use is not recommended in any patient [2, 4]. Acetaminophen provides little-to-no efficacy for OA-related pain, and long-term use of opioids is not recommended due to serious safety concerns and a lack of long-term efficacy in chronic pain conditions [2, 4–6]. Indeed, the lack of sufficient pain management options when the risks of NSAIDs and COX-2 inhibitors were increasingly recognized may have contributed to an increase in opioid prescribing for arthritic conditions such as OA [7].

Nerve growth factor (NGF) is associated with pro-nociceptive functions implicated in the pathogenesis of pain including promotion of peripheral and central sensitization and enhanced local neuronal sprouting at sites of inflammation, within the dorsal root ganglion, and, possibly, within the dorsal horn [8]. Tanezumab is a monoclonal antibody against NGF under investigation for the treatment of the signs and symptoms of moderate-to-severe OA for whom other analgesics are ineffective, contraindicated, or not tolerated. In short-term studies of OA, intravenous (IV) tanezumab has typically provided greater improvements in pain and function compared with NSAIDs or opioids and was generally well-tolerated [9].

A recent phase 3, randomized, double-blind, active-controlled, multicenter, parallel-group study investigated the long-term safety and efficacy of subcutaneous (SC) tanezumab compared with NSAIDs in participants with moderate-to-severe OA of the hip or knee [10]. The overall safety profile of tanezumab was consistent with previous shorter-term OA studies, and tanezumab was associated with significantly more joint safety events than NSAIDs in a dose-dependent fashion. Both tanezumab and NSAIDs improved self-reported pain and physical function at 16-week and 56-week timepoints. Here, we focus our evaluation on details regarding the time course and maintenance of these efficacy responses over 1 year of treatment with tanezumab or NSAIDs and characterize the clinical meaningfulness, patient satisfaction, and treatment preferences associated with these responses.

On October 26, 2021, Pfizer Inc. and Eli Lilly and Company announced the discontinuation of the tanezumab global clinical development program as a result of the outcomes of regulatory reviews of tanezumab for the treatment of osteoarthritis pain by the U.S. Food and Drug Administration and European Medicines Agency [11, 12].

## Methods

### Study design

This phase 3, randomized, double-blind, double-dummy, active-controlled (NSAID), parallel-group study (ClinicalTrials.gov: NCT02528188) was conducted at 446 clinical research, specialist/general practice, or hospital sites in the USA, Europe, Latin America, and Asia-Pacific region from July 2015 to February 2019.

The study consisted of a screening period of up to 37 days (including a 2- to 30-day washout phase for prohibited medications and an initial pain assessment period [IPAP] of 7 days prior to baseline), a 56-week double-blind treatment period, and a 24-week safety follow-up period that began 8 weeks after final SC injection. The co-primary efficacy endpoints were change in Western Ontario and McMaster Universities Osteoarthritis Index (WOMAC© 1996 Nicholas Bellamy. WOMAC® is a registered trademark of Nicholas Bellamy [CDN, EU, USA]) Pain, WOMAC Physical Function, and Patient Global Assessment of Osteoarthritis (PGA-OA) at week 16 for each tanezumab group versus the NSAID group. The full methodology for this study has been reported previously [10].

### Study sample

Eligibility criteria included age ≥ 18 years, body mass index (BMI) ≤ 39 kg/m^2^, American College of Rheumatology classification criteria for hip or knee OA, a Kellgren-Lawrence (KL) grade ≥ 2 in index joint (most painful hip or knee) as confirmed by the central reader, WOMAC Pain and WOMAC Physical Function scores of ≥ 5 at baseline, and a PGA-OA rating of “fair,” “poor,” or “very poor” at baseline. A documented history of inadequate pain relief or intolerance to standard OA pain treatment (inadequate pain relief with acetaminophen, tramadol, or non-tramadol opioid analgesic; intolerance or contraindication to tramadol or non-tramadol opioid; or unwillingness to take a non-tramadol opioid analgesic) was also required. Finally, participants were required to be receiving treatment with a qualifying NSAID regimen averaging ≥ 5 days per week during the 30 days prior to the screening visit. Exclusion criteria included radiographic evidence in any joint of prespecified bone or joint conditions (e.g., destructive arthropathy characteristic of rapidly progressive OA [RPOA], atrophic OA, subchondral insufficiency fracture, primary osteonecrosis, or pathologic or stress fracture) as determined by a central musculoskeletal radiologist; history of osteonecrosis or osteoporotic fracture, or significant trauma or surgery to a knee, hip, or shoulder within the previous year; history or presence of clinically significant neurological, cardiovascular, or psychiatric disorders, cancer (except certain skin cancers), fibromyalgia, or sciatica; oral or intramuscular corticosteroid within 30 days or intra-articular corticosteroid injection in the index joint within 12 weeks or in any other joint within approximately 30 days of the IPAP; or intraarticular hyaluronic acid injection in the index joint within 30 days or long-acting hyaluronic acid formulation injection in the index joint within approximately 18 weeks of the IPAP.

### Interventions

Participants received a stable, open-label, dosing regimen of oral NSAID (naproxen 500 mg twice daily [BID], celecoxib 100 mg BID, or diclofenac extended-release 75 mg BID) for at least the last 2 weeks of the screening period (participants receiving stable naproxen, celecoxib, or diclofenac prior to screening received the same NSAID during screening, while those receiving other stable NSAIDs prior to screening were assigned to naproxen, celecoxib, or diclofenac at the investigator’s discretion during screening). After the screening period ended, participants were randomized in a 1:1:1 manner to tanezumab 2.5 mg SC plus oral placebo, tanezumab 5 mg SC plus oral placebo, or oral NSAID (the same NSAID regimen received during screening) plus SC placebo. SC study medication was administered by site staff every 8 weeks (up to 7 doses) through week 56. Participants self-administered oral study medication BID for up to 56 weeks.

Except for the 24-h period prior to study visits, the rescue medication (acetaminophen) was permitted for participants requiring additional pain relief at doses ≤ 3000 mg/day for up to 3 days/week up to week 16 and then daily after week 16. The use of non-assigned NSAIDs was prohibited through week 64, but occasional use of other analgesics was permitted for self-limiting conditions unrelated to OA.

To continue receiving SC study medication beyond week 16, participants had to meet pre-specified efficacy criteria including a ≥ 15% reduction in WOMAC Pain subscale score from baseline to weeks 2, 4, or 8, and a ≥ 30% reduction in WOMAC Pain score from baseline in the index joint at week 16. Participants not meeting both these criteria were discontinued (reason defined as “met pain criteria for discontinuation”) from the treatment period at week 16 and entered a 24-week early termination follow-up period.

### Efficacy measures

Participants completed WOMAC and PGA-OA questionnaires at baseline and weeks 2, 4, 8, 16, 24, 32, 40, 48, 56, and 64 [13]. The 1-item PGA-OA assesses current overall OA status on a 5-point Likert scale from 1 = very good to 5 = very poor. Change from baseline was analyzed and presented for these measures at each assessment timepoint up to week 56; Week 64 data were only used to assess response following treatment discontinuation.

Participants used an electronic diary (eDiary) to assess average pain in the index joint over the past 24 h (on a numeric rating scale [NRS] from 0 = no pain to 10 = worst possible pain) daily to week 16 and then weekly until week 80. Change from baseline was analyzed for days 1–7 and for weeks 1, 2, 3, 4, 6, 8, 10, 12, 16, 20, 24, 32, 40, 48, and 56; Week 64 data (not shown) were only used to assess response following treatment discontinuation.

The proportion of participants achieving ≥ 30% (moderate), ≥ 50% (substantial), ≥ 70%, or ≥ 90% improvement from baseline in WOMAC Pain, WOMAC Physical Function, and the proportion meeting Outcome Measures in Rheumatology-Osteoarthritis Research Society International (OMERACT-OARSI) treatment response criteria (see Fig. 5 footnote for definition) was analyzed at weeks 2, 4, 8, 16, 24, 32, 40, 48, and 56 [14–16]. The proportion of patients achieving ≥ 30%, ≥ 50%, ≥ 70%, or ≥ 90% improvement from baseline in average pain in the index joint was analyzed at weeks 1, 2, 3, 4, 6, 8, 10, 12, 16, 20, 24, 32, 40, 48, and 56. To characterize time course and long-term maintenance of effect, these responder data are presented for weeks 2, 4, 8, 16, and 56. The thresholds of improvement of ≥ 30% and ≥ 50% were evaluated since they represent moderate and substantial, respectively, thresholds of clinically meaningful improvement in patients with chronic pain [14]. In order to provide a more complete assessment of treatment efficacy, we also evaluated thresholds of ≥ 70% and ≥ 90% to explore whether even greater levels of improvement can be achieved with tanezumab or NSAID treatment.

Minimum Clinically Important Improvement (MCII) and Patient Acceptable Symptom State (PASS) are categorical patient-reported outcomes that aim to define treatment response at the individual/patient level. MCII is defined as the smallest change in measurement that signifies an important improvement in patient symptoms and PASS is defined as the value beyond which participants consider themselves well. Both endpoints were assessed at weeks 16 and 56 and were based on objective response criteria (changes in average pain in the index joint, WOMAC Physical Function, and PGA-OA scores; see footnote to Supplementary Fig. 1 for explanation of response criteria). The proportion of participants achieving an early sustained MCII or PASS response, defined as meeting the respective criteria at weeks 4 through 16, was also assessed. The week 4 timepoint was chosen based on tanezumab pharmacokinetics (achievement of steady-state) and since it was the first timepoint where a large proportion of participants experienced meaningful (≥ 30%) symptom improvement, and week 16 was chosen since it was the pre-specified primary efficacy timepoint and included limits on rescue medication up to this timepoint.

Participants recorded rescue medication use in the eDiary daily through week 16 and then weekly through week 80. The incidence of rescue medication use and the mean/median number of days of rescue medication use per week were analyzed and presented for weeks 2, 4, 8, 16, 24, 32, 40, 48, and 56. The amount (mg) of rescue medication taken was analyzed for weeks 2, 4, 8, and 16 only, since the amount of rescue medication was not assessed daily after week 16.

The modified Patient-Reported Treatment Impact (mPRTI) questionnaire and the Treatment Satisfaction Questionnaire for Medication V.II (TSQM) were completed at weeks 16 and 56 to assess study treatment preference and satisfaction.

### Safety measures

Musculoskeletal and neurological examinations, monitoring of AEs, and review of joint pain scores were conducted by investigators throughout the study. Radiographs of the bilateral hips, knees, and shoulders (obtained at screening and weeks 24, 56, and 80) were evaluated by trained central readers to monitor for possible joint safety events. Possible joint safety events, identified post-screening, were adjudicated by a blinded external committee of experts, and prespecified joint safety events were included in a composite joint safety endpoint (Supplementary Text 1). Full details of the safety measures used in the study and their results have been published previously [10].

### Statistical methods

The sample size was established, primarily, to have a high probability of observing participants with any component of the primary composite joint safety endpoint, assuming a low event rate, rather than for efficacy assessments. However, a sample size of approximately 1000 participants per group was estimated to provide 76% power for comparison of the co-primary efficacy endpoints (change in WOMAC Pain, WOMAC Physical Function, and PGA-OA at week 16) for both tanezumab groups versus the NSAID group. The intent to treat the population (all randomized participants who received ≥ 1 dose of SC study medication) was the primary analysis set for efficacy and safety.

Changes from baseline in WOMAC Pain, WOMAC Physical Function, and PGA-OA were prespecified co-primary (week 16) or secondary (other study weeks) efficacy endpoints. All other assessments were prespecified secondary endpoints except for analyses of responder rates for MCII, PASS, and average pain in the index joint, which were exploratory post hoc endpoints.

As reported previously, the co-primary and key secondary (proportion of participants with ≥ 50% improvement in WOMAC Pain at week 16) endpoints were included in a multiple testing procedure to control the family-wise type 1 error, using a graphical gatekeeping strategy [10]. Since the tanezumab 5 mg dose failed to achieve statistical significance for one co-primary endpoint (PGA-OA at week 16), hypothesis testing of the tanezumab 2.5 mg dose for the co-primary endpoints and of both doses for the key secondary endpoint could not be performed [10]. Since the objective of the current manuscript is to evaluate the time course of treatment effect and clinical importance of response using a mixture of primary, key secondary, other secondary, and post hoc endpoints, data in this manuscript are presented with unadjusted *p* values for directional guidance and consistency across timepoints.

An analysis of covariance (ANCOVA) model was used for the analysis of change from baseline in WOMAC Pain, WOMAC Physical Function, PGA-OA, and average pain in the index joint scores with a multiple imputation approach for missing data (dependent on the reason for missing data). Responder rates for WOMAC Pain, WOMAC Physical Function, average pain in the index joint, OMERACT-OARSI, MCII, and PASS were analyzed using logistic regression with a mixed baseline-observation-carried-forward (BOCF)/last-observation-carried-forward (LOCF) approach to missing data. Rescue medication use was analyzed using logistic regression (incidence) or negative binomial (amount and number of days) models with a LOCF approach to missing data. mPRTI (treatment preference) scores were analyzed using a Cochran-Mantel-Haenszel test (stratified by combinations of index joint, highest KL grade, and NSAID) using observed data. TSQM (treatment satisfaction) scores were analyzed using an ANCOVA model with observed data.

## Results

### Patient disposition and baseline characteristics

Of 3021 participants randomized, 2996 received ≥ 1 dose of SC study medication: tanezumab 2.5 mg = 1002, tanezumab 5 mg = 998, and NSAID = 996 (diclofenac = 193 [19.4%], celecoxib = 321 [32.2%], and naproxen = 482 [48.4%]). Patient characteristics were similar across groups: female = 63.6–66.5%, mean age = 60.3–61.2 years, proportion aged ≥65 years = 32.1–36.2%, and the knee as the index joint = 84.9–85.5%. The mean (standard deviation, [SD]) scores at baseline were WOMAC Pain = 7.0 (1.1) in all groups; WOMAC Physical Function = 7.1 (1.1) in both tanezumab groups and 7.0 (1.1) in the NSAID group; and PGA-OA = 3.5 (0.6) in both tanezumab groups and 3.4 (0.6) in the NSAID group. Prestudy analgesic treatment history is shown in Supplementary Table 1.

Overall, 1807 (60.3%) of treated participants received SC medication at week 16 and 1312 (43.8%) completed the 56-week treatment period. Of those participants completing the 56-week treatment period, 1222 (93.1%) also completed the safety follow-up period (to week 80). The most common reasons for discontinuations during the treatment period were “participant met pain criteria for discontinuation”(i.e., did not achieve reductions from baseline in WOMAC Pain of ≥ 15% at week 2, 4, or 8, *and* of ≥ 30% at week 16; overall = 21.7%, tanezumab 2.5 mg = 22.4%, tanezumab 5 mg = 20.7%, NSAID = 22.1%); “other” (overall = 9.5%, tanezumab 2.5 mg = 10.0%, tanezumab 5 mg = 9.8%, NSAID = 8.8%); “AE” (overall = 7.9%, tanezumab 2.5 mg = 7.4%, tanezumab 5 mg = 10.4%, NSAID = 5.8%); and “insufficient clinical response” (i.e., any participant- or investigator-directed discontinuation related to inadequate efficacy not specifically due to the WOMAC Pain criteria described above; overall = 7.1%, tanezumab 2.5 mg = 6.0%, tanezumab 5.0 mg = 6.3%, NSAID = 9.1%). The category “other” was used for patients who discontinued due to reasons that prevented them from further participation, such as a move or change in life or work circumstances.

Use of NSAIDs, other than study treatment, was low across all treatment groups for each 8-week SC dosing interval up to week 56; the mean number of days was < 1.5, and the median number of days was 0 for each interval.

### Efficacy

#### WOMAC subscales and PGA-OA

**Fig. 1:**
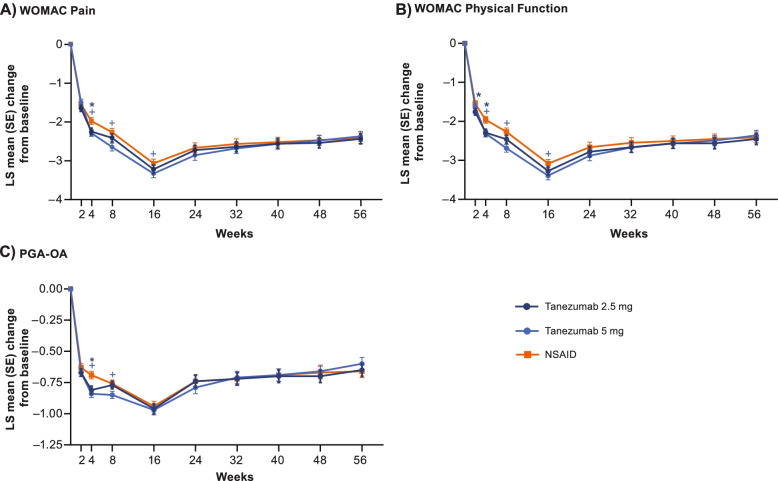
Change in WOMAC Pain, WOMAC Physical Function, and PGA-OA. LS mean (SE) changes, from baseline, in WOMAC Pain (**A**), WOMAC Physical Function (**B**), and PGA-OA (**C**) scores are shown over the 56-week treatment period. *Unadjusted *p* ≤ 0.05 for tanezumab 2.5mg versus NSAID. **+**Unadjusted *p* ≤ 0.05 for tanezumab 5mg versus NSAID. LS, least squares; NSAID, nonsteroidal anti-inflammatory drug; PGA-OA, Patient’s Global Assessment of Osteoarthritis; SE, standard error; WOMAC, Western Ontario and McMaster Universities Osteoarthritis Index

All treatment groups improved WOMAC Pain, WOMAC Physical Function, and PGA-OA scores over the 56-week treatment period relative to baseline (Fig. 1A–C). Across all groups, improvements for each outcome generally occurred between weeks 2 to 16 and then slightly decreased from week 16 to 24 before stabilizing from weeks 24 to 56. There was no evidence of a rebound effect (increase above baseline) at week 64 (16 weeks after the last dose of SC study medication) in any treatment group for any outcome measure (Supplementary Table 2).

Changes from baseline in WOMAC Pain, WOMAC Physical Function, and PGA-OA were generally similar across treatment groups, though improvements were sometimes greater (unadjusted *p* ≤ 0.05) with tanezumab than with NSAID at timepoints on or before week 16 (Fig. 1A–C).

#### Average pain

**Fig. 2:**
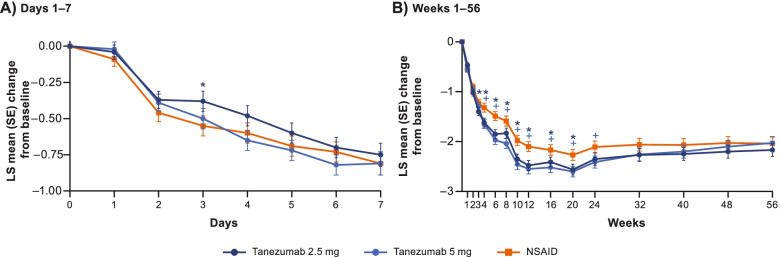
Change in average pain in the index joint. LS mean (SE) changes, from baseline, in average pain in the index joint scores are shown over the first 7 days (**A**) and full 56 weeks (**B**) of treatment. *Unadjusted *p* ≤ 0.05 for tanezumab 2.5mg versus NSAID. **+**Unadjusted *p* ≤ 0.05 for tanezumab 5mg versus NSAID. LS, least squares; NSAID, nonsteroidal anti-inflammatory drug; SE, standard error

All treatment groups provided sustained improvement in average pain in the index joint over the 56-week treatment period relative to baseline (Fig. 2). However, improvements were greater (unadjusted *p* ≤ 0.05) for tanezumab at some time-points (2.5 mg from weeks 3–20; 5 mg from weeks 4–24) than for NSAID.

#### WOMAC Pain, WOMAC Physical Function, and average pain responder status

**Fig. 3:**
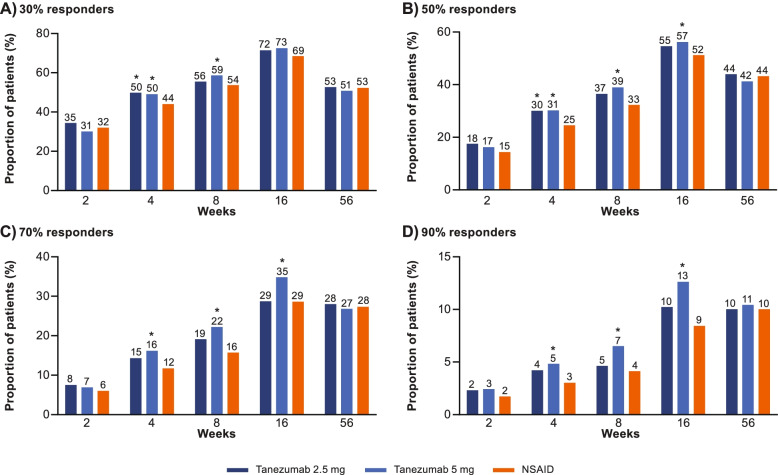
WOMAC Pain responders. The proportion of patients achieving ≥30% (**A**), ≥50% (**B**), ≥70% (**C**), or ≥90% (**D**) reductions from baseline in WOMAC Pain at weeks 2, 4, 8, 16, and 56 is shown. Proportions shown above each bar are rounded to the nearest percent. *Unadjusted *p* ≤ 0.05 versus NSAID. NSAID, nonsteroidal anti-inflammatory drug; WOMAC, Western Ontario and McMaster Universities Osteoarthritis Index

**Fig. 4:**
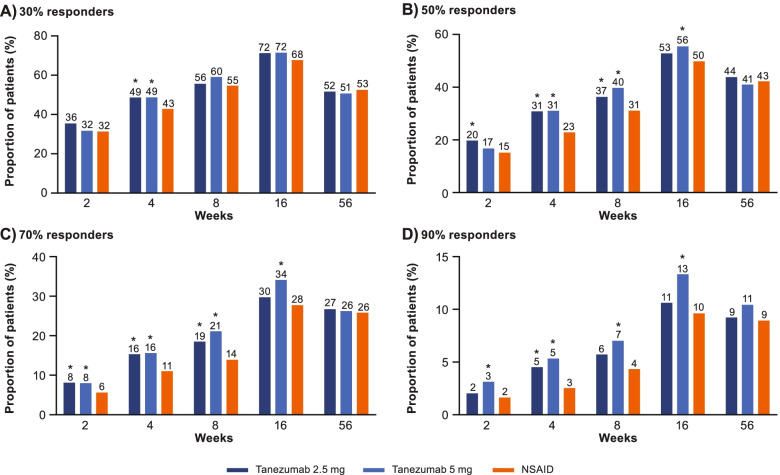
WOMAC Physical Function responders. The proportion of patients achieving ≥30% (**A**), ≥50% (**B**), ≥70% (**C**), or ≥90% (**D**) reductions from baseline in WOMAC Physical Function at weeks 2, 4, 8, 16, and 56 is shown. Proportions shown above each bar are rounded to the nearest percent. *Unadjusted *p* ≤ 0.05 versus NSAID. NSAID, nonsteroidal anti-inflammatory drug; WOMAC, Western Ontario and McMaster Universities Osteoarthritis Index

**Fig. 5:**
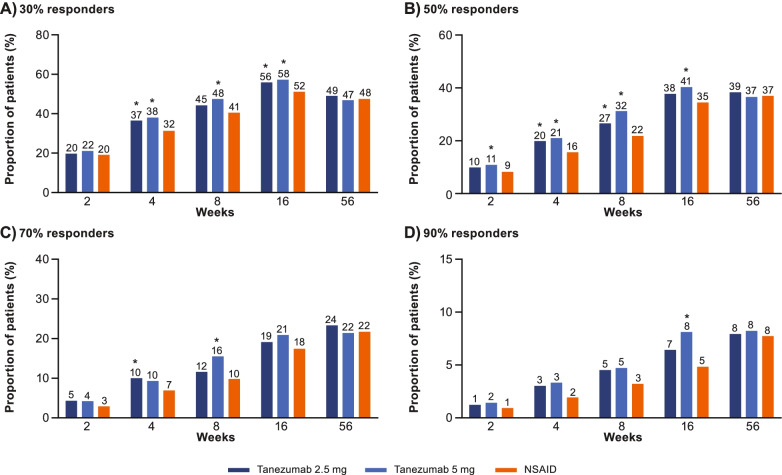
Average pain in the index joint responders. The proportion of patients achieving ≥30% (**A**), ≥50% (**B**), ≥70% (**C**), or ≥90% (**D**) reductions from baseline in average pain in the index joint at weeks 2, 4, 8, 16, and 56 is shown. Proportions shown above each bar are rounded to the nearest percent. *Unadjusted *p* ≤ 0.05 versus NSAID. NSAID, nonsteroidal anti-inflammatory drug

The proportion of participants achieving ≥ 30%, ≥ 50%, ≥ 70%, and ≥ 90% improvement, from baseline, in WOMAC Pain at weeks 2, 4, 8, 16, and 56 is shown in Fig. 3A–D. Across all groups, the proportion of participants achieving moderate (≥ 30%) improvement in WOMAC Pain was 68.9–72.9% at week 16 and 51.2–53.1% at week 56. The proportion of participants achieving substantial (≥ 50%) improvement in WOMAC Pain was 51.5–56.5% at week 16 and 41.5–44.3% at week 56. The proportion of participants achieving ≥ 30%, ≥ 50%, ≥ 70%, and ≥ 90% improvement in WOMAC Pain was larger (unadjusted *p* ≤ 0.05) in the tanezumab groups than in the NSAID group at some timepoints, typically on or before week 16. Similar findings were observed for WOMAC Physical Function (Fig. 4A–D) and for average pain in the index joint (Fig. 5A–D).

#### OMERACT-OARSI responder status

**Fig. 6:**
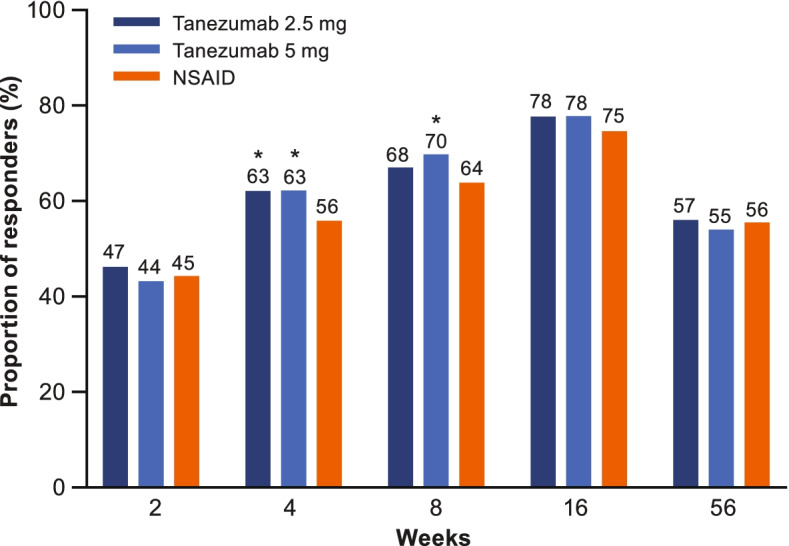
OMERACT-OARSI responders. The proportion of patients meeting OMERACT-OARSI responder criteria at weeks 2, 4, 8, 16, and 56 is shown. Response criteria were defined as a within-patient improvement from baseline of (i) ≥ 50% and ≥ 2 points in either WOMAC Pain or Physical Function or (ii) ≥ 20% and ≥ 1 point in two of the following: WOMAC Pain, WOMAC Physical Function, or PGA-OA. Proportions shown above each bar are rounded to the nearest percent. *Unadjusted *p* ≤ 0.05 versus NSAID. NSAID, nonsteroidal anti-inflammatory drug; OMERACT-OARSI, Outcome Measures in Arthritis Clinical Trials-Osteoarthritis Research Society International; PGA-OA, Patient’s Global Assessment of Osteoarthritis; WOMAC, Western Ontario and McMaster Universities Osteoarthritis Index

Across all treatment groups, the proportion of participants meeting criteria for OMERACT-OARSI responder status over the course of the treatment period was 43.7–46.7% at week 2, 75.1–78.3% at week 16, and 54.5–56.5% at week 56 (Fig. 6). The proportion of participants meeting response criteria was greater (unadjusted *p* ≤ 0.05), compared with NSAID, in both tanezumab groups at week 4 and in the tanezumab 5 mg group at Week 8.

#### MCII and PASS

A similar proportion of participants achieved MCII at week 16 (tanezumab 2.5 mg = 48.3%, tanezumab 5 mg = 48.0%, NSAID = 44.9%) and week 56 (tanezumab 2.5 mg = 39.3%, tanezumab 5 mg = 37.0%, NSAID = 40.1%) in the tanezumab and NSAID groups (Supplementary Fig. 1A). The proportion of participants with an early sustained MCII response from week 4 through week 16 was greater (unadjusted *p* ≤ 0.05) with both doses of tanezumab (2.5 mg = 22.6%, 5 mg = 23.7%) than with NSAID (18.0%) (Supplementary Fig. 1B). The proportion of participants achieving PASS was similar across groups at week 16 (tanezumab 2.5 mg = 28.3%, tanezumab 5 mg = 29.6%, NSAID = 27.2%) and week 56 (tanezumab 2.5 mg = 27.5%, tanezumab 5 mg = 25.0%, NSAID = 26.2%) (Supplementary Fig. 1C). The proportion of participants with an early sustained PASS response from week 4 through week 16 was greater (unadjusted *p* ≤ 0.05) with tanezumab 5 mg (9.9%) than with NSAID (6.9%) (Supplementary Fig. 1D). An early sustained PASS response from week 4 through week 16 was achieved in 8.8% of participants in the tanezumab 2.5 mg group.

#### Use of rescue medication

The incidence of rescue medication use and the mean/median number of days of rescue medication use per week were similar in the tanezumab and NSAID groups from week 2 to week 56 (data not shown). The amount (mg) of rescue medication used was lower (unadjusted *p* ≤ 0.05) in the tanezumab 5 mg group than in the NSAID group at weeks 4 and 8 (Supplementary Table 3).

#### Patient-reported treatment preference (mPRTI) and treatment satisfaction (TSQM)

A greater (unadjusted *p* ≤ 0.05) proportion of participants in the tanezumab 5 mg group (80.3%) slightly or definitely preferred the drug they received in the study to previous OA treatment they had received compared with patients in the NSAID group (73.6%) at week 16. A greater (unadjusted *p* ≤ 0.05) proportion of participants receiving tanezumab also reported that they would be willing to use the drug they received in the study for OA pain in the future (both doses at week 16, 2.5 mg dose at week 56) (Supplementary Table 4). Least squares (LS) mean (standard error [SE]) global treatment satisfaction scores were better (unadjusted *p* ≤ 0.05) in both tanezumab groups compared with NSAID at week 16 (tanezumab 2.5 mg = 70.3 [1.0], tanezumab 5 mg = 70.7 [1.0], NSAID = 67.1 [1.0]) though there was little difference across groups at week 56 (Supplementary Table 5).

### Safety

Safety findings from this study have been published previously [10]. Briefly, the incidence of AEs, serious AEs (SAEs), and treatment discontinuations due to AEs (but continuing in the study) during the 56-week treatment period was highest with tanezumab 5 mg (AEs = 67.1%, SAEs = 8.0%, discontinuations = 8.8%) and similar between the tanezumab 2.5 mg (AEs = 62.8%, SAEs = 5.1%, discontinuations = 5.3%) and NSAID (AEs = 60.3%, SAEs = 4.6%, discontinuations = 5.2%) groups. The most common SAEs were musculoskeletal or connective tissue events in tanezumab groups (Supplementary Table 6). AEs of abnormal peripheral sensation were more frequent in both tanezumab groups (2.5 mg = 6.2%, 5 mg = 9.0%) than in the NSAID group (4.6%), but a majority of these events resolved by the end of study (tanezumab 2.5 mg = 72.6%, tanezumab 5 mg = 76.7%, NSAID = 87.0%) and neurologic diagnoses were similar across all groups.

Observation time-adjusted rates per 1000 patient-years (95% confidence interval [95% CI]) of the composite joint safety endpoint were higher with tanezumab (2.5 mg = 38.3 [28.0, 52.5], unadjusted *p* = 0.001; 5 mg = 71.5 [56.7, 90.2], unadjusted *p* < 0.001) than with NSAID (14.8 [8.9, 24.6]). Likewise, observation time-adjusted rates of total joint replacement surgery were higher with tanezumab 2.5 mg (51.8 [39.6, 67.9], unadjusted *p* =0.003) and 5 mg (79.7 [64.0, 99.2], unadjusted *p* < 0.001) compared with NSAID (25.7 [17.5, 37.7]).

## Discussion

In this randomized trial primarily designed to assess long-term safety in participants with moderate-to-severe OA of the knee or hip, a history of inadequate response to standard OA analgesics, and on a stable dose of oral NSAIDs prior to enrollment, improvements in pain and function were observed early (by week 1 or 2, depending on the time of first assessment for a particular instrument) and were sustained throughout the 56-week treatment period in response to treatment with SC tanezumab or oral NSAID. Improvements were clinically meaningful in a large proportion of participants and were supported by assessments of patient preference and satisfaction with study treatment over 56 weeks, complementing previous reports of tanezumab efficacy at 16 weeks [17, 18] and 24 weeks [19] in patients with moderate-to-severe OA.

The proportion of participants achieving ≥ 30% reduction in WOMAC Pain or Physical Function, meeting OMERACT-OARSI response criteria, and achieving MCII or PASS at weeks 16 and 56 provide evidence for sustained and clinically meaningful efficacy across all treatment groups in the current study. Further, the proportion of participants achieving clinically meaningful response may be underestimated in our analysis since MCII and PASS definitions were based on previous 4-week studies that, compared with the current study, enrolled participants with less severe OA-related pain (≥30 mm on a scale from 0 to 100mm) and did not require a history of inadequate response to standard analgesics [20, 21]. Likewise, though a reduction in pain of ≥30% is considered a benchmark for “moderate” clinically meaningful improvement, lower reductions in chronic pain (10–20%) may be considered meaningful to some patients [15].

Across all efficacy measures, the magnitude of improvements observed with tanezumab was at least similar to, and sometimes greater (unadjusted *p* ≤ 0.05) than, NSAID at timepoints up to and including week 16. Efficacy was generally more similar (with a few exceptions) between the tanezumab and NSAID groups after week 16. Exclusion of inadequate responders using prespecified criteria at week 16, and other discontinuations due to lack of efficacy, may have contributed to this finding in the following ways. First, a consequence of these discontinuations is that the trial is enriched with responders at later timepoints in all groups, inducing a selection bias and making it difficult to detect treatment differences across groups. Second, all discontinuations (related to efficacy or tolerability) contributed to a reduction in the amount of observed data at later timepoints; less than half of treated participants had efficacy data available at week 56. Although a BOCF approach was utilized consistently across treatment groups to account for missing data due to efficacy-related study discontinuation, this approach tends to underestimate mean changes from baseline and makes detection of treatment differences more difficult (this approach may also explain some of the slight loss of efficacy observed in all treatment groups from weeks 16 to 24). Discontinuation of non-responders limits comparisons of efficacy between the tanezumab and NSAID groups at timepoints beyond week 16 but was incorporated into study design, since the primary objective was to assess long-term safety, to limit exposure and minimize potential AEs in patients who did not experience an efficacy response. Increased use of rescue medication was allowed after week 16, and this could have also potentially confounded efficacy assessment of study treatments and comparisons across groups at later timepoints. Though the proportion of patients using rescue medication (and the mean number of days rescue medication was used) was similar across groups after week 16, the amount (mg/day) of rescue medication used was not assessed daily after week 16 and this limits an evaluation of whether any potential differences in rescue medication use contributed to the lack of treatment difference in the tanezumab and NSAID groups at later timepoints.

Since participants received stable NSAID treatment prior to baseline in this study, there should have been little-to-no efficacy response observed in the NSAID group. However, substantial efficacy was observed in the blinded NSAID group, suggesting a greater than expected “placebo-like” response based on previous NSAID-controlled studies of tanezumab [22, 23]. Supporting this theory, the proportion of participants discontinuing treatment for not meeting prespecified efficacy criteria at week 16 (21.7% across all treatment arms) was lower than expected based on projections (35–45%) from earlier phase 3 studies of tanezumab. This “placebo-like” response may have also contributed to smaller than expected differences in WOMAC Pain among the tanezumab and NSAID treatment groups. While a greater than expected “placebo-like” response was observed with the WOMAC Pain subscale, the NSAID response was more typical (smaller) using the daily average pain NRS and significant treatment differences were observed for both tanezumab groups versus NSAID at multiple timepoints from weeks 4 to 20. Efforts to minimize placebo response (e.g., patient education on the topic and management of treatment expectations) were not specifically addressed in the study design. Alternatively, it is also possible that participants were not taking NSAIDs in a truly stable basis prior to trial enrollment, resulting in a greater-than-expected response when stable NSAID study treatment was initiated. An unexplained increasing placebo response, however, has been observed over the course of the switch from phase 2 IV to phase 3 SC studies in the tanezumab development program, and a similar phenomenon has been observed in late-stage clinical development programs of other analgesics, possibly related to the greater patient expectation of treatment response in late stages of clinical development programs [24, 25].

It should be noted that improvements observed for average pain in the index joint (using the eDiary NRS) were typically lower than those observed for the WOMAC Pain subscale, which may reflect inherent differences in the assessment tools or how they were administered. The eDiary NRS was completed by the patient at home and consisted of a single question on pain severity over the previous 24 h, while the WOMAC Pain was completed at clinic visits (and screening to determine trial eligibility) and assessed five different aspects of “the pain you felt” over the last 48 h. It is possible that the discrepancy between eDiary NRS and WOMAC Pain scores may be partly due to unconscious cues associated with clinic visits.

Over the 56-week treatment period, tanezumab 2.5 mg and 5 mg doses yielded generally similar results across endpoints. There were some instances, however, where tanezumab 5 mg may have provided better efficacy than tanezumab 2.5 mg. At weeks 8 and 16, for example, improvements in WOMAC Pain and WOMAC Physical Function were statistically greater with tanezumab 5 mg, but not tanezumab 2.5 mg, compared to NSAIDs. Direct comparisons, however, were not performed between tanezumab dose groups and firm conclusions on possible differences in efficacy cannot be made. Though tanezumab 5 mg may have demonstrated moderately better efficacy than tanezumab 2.5 mg at some time points, it should be noted that the 5 mg dose exhibited a less favorable AE profile, including joint-related AEs, than the 2.5 mg dose.

## Conclusion

This study is the longest assessment of tanezumab in participants with OA to date (56-week treatment and 24-week safety follow-up), which is notable since long-term trial data on OA treatments, including NSAIDs, is generally lacking [26]. Though firm conclusions on long-term efficacy (and comparisons of efficacy across groups) are limited due to elements of trial design (e.g., enrichment for responders, BOCF approach to missing data), this study suggests that treatment with SC tanezumab (2.5 mg and 5 mg) or oral NSAIDs provides early and sustained (up to 56 weeks) efficacy relative to baseline in participants with moderate-to-severe OA of the knee or hip and a history of inadequate response to standard OA analgesics. Improvements in pain and function were clinically meaningful in a substantial proportion of participants. Though tanezumab was well-tolerated in most participants, dose-dependent joint safety events occurred more frequently with tanezumab than with NSAID and should be taken into account when assessing tanezumab’s overall risk/benefit profile.

## Supplementary Information

**Additional file 1: Supplementary Text 1.** Composite joint safety endpoint. Text describing how joint safety events were monitored and adjudicated.

**Additional file 2: Supplementary Table 1.** Pre-study OA analgesic treatment history in randomized participants who received at least 1 SC dose. Table showing analgesic OA treatments used by participants in each treatment group prior to trial enrollment.

**Additional file 3: Supplementary Table 2.** Change in WOMAC Pain, WOMAC Physical Function, and PGA-OA at weeks 56 and 64. Table showing the change from baseline in WOMAC Pain, WOMAC Physical Function, and PGA-OA scores for each treatment group at Weeks 56 and 64 using only observed data.

**Additional file 4: Supplementary Table 3.** Amount (mg) of rescue medication use per week. Table showing the amount of rescue medication used in each treatment group at Weeks 2, 4, 8, and 16.

**Additional file 5: Supplementary Table 4.** Patient-reported treatment preference for study medication using mPRTI. Table summarizing treatment preference scores in each treatment group at Weeks 16 and 56.

**Additional file 6: Supplementary Table 5.** Global treatment satisfaction with study medication using the TSQM. Table summarizing treatment satisfaction scores in each treatment group at Weeks 16 and 56.

**Additional file 7: Supplementary Table 6.** Serious musculoskeletal and connective tissue AEs during the 56-week treatment period. Table summarizing serious musculoskeletal and connective tissue AEs in each treatment group during the 56-week treatment period.

**Additional file 8: Supplementary Fig. 1.** The proportion of participants with a clinically important improvement. Panel **A** shows the proportion of participants achieving MCII at Weeks 16 and 56. MCII was defined as improvements from baseline in average pain in the index joint of ≥1.99 (knee) or ≥ 1.53 (hip), in WOMAC Physical Function of ≥ 0.91 (knee) or ≥ 0.79 (hip), and in PGA-OA of ≥ 1 point/category. Panel **B** shows the proportion of participants achieving a sustained MCII response from Weeks 4 through 16. Panel **C** shows the proportion of participants achieving PASS at Weeks 16 and 56. PASS was defined as an average pain score in the index joint of ≤ 3.23 (knee) or ≤ 3.50 (hip), a WOMAC Physical Function score of ≤ 3.10 (knee) or ≤ 3.44 (hip), and a PGA-OA score of “good” or “very good”. Panel **D** shows the proportion of participants achieving a sustained PASS response from Weeks 4 through 16. Proportions shown above each bar are rounded to the nearest percent. The proportions of participants meeting specified response criteria were analyzed using logistic regression analyses. *Unadjusted *p* ≤ 0.05 versus NSAID. *MCII* Minimum Clinically Important Improvement, *PASS* Patient Acceptable Symptom State, *PGA-OA* Patient’s Global Assessment of osteoarthritis, *WOMAC* Western Ontario and McMaster Universities Osteoarthritis Index.

## Data Availability

Upon request, and subject to review, Pfizer will provide the data that support the findings of this study. Subject to certain criteria, conditions, and exceptions. Pfizer may also provide access to the related individual de-identified participant data. See https://www.pfizer.com/science/clinical-trials/trial-data-and-results for more information.

## Abbreviations

AEs: Adverse events
ANCOVA: Analysis of covariance
BID: Twice daily
BOCF: Baseline-observation-carried-forward
CI: Confidence interval
eDiary: Electronic diary
IV: Intravenous
KL: Kellgren-Lawrence
LOCF: Last-observation-carried-forward
MCII: Minimum Clinically Important Improvement
mPRTI: Patient-Reported Treatment Impact Assessment-modified
NGF: Nerve growth factor
NRS: Numeric rating scale
NSAIDs: Nonsteroidal anti-inflammatory drugs
OMERACT-OARSI: Outcome Measures in Rheumatology-Osteoarthritis Research Society International
OA: Osteoarthritis
PASS: Patient Acceptable Symptom State
PGA-OA: Patient’s Global Assessment of OA
RPOA: Rapidly progressive osteoarthritis
SAEs: Serious adverse events
SC: Subcutaneous
SD: Standard deviation
TSQM: Treatment Satisfaction Questionnaire for Medication V.II
WOMAC: Western Ontario and McMaster Universities Osteoarthritis Index
