# Supplementary material for: Observed efficacy and clinically important improvements in participants with osteoarthritis treated with subcutaneous tanezumab: results from a 56-week randomized NSAID-controlled study
Source: Arthritis Res Ther. 2022 Mar 29;24:78. doi: 10.1186/s13075-022-02759-0 (PMC8966257; doi:10.1186/s13075-022-02759-0)
Supplement: Supplementary file 1 — Additional file 1: Supplementary Text 1. Composite joint safety endpoint. Text describing how joint safety events were monitored and adjudicated. [file 13075_2022_2759_MOESM1_ESM.docx]

**Supplementary Text 1. Composite joint safety endpoint**

A blinded Adjudication Committee, comprising external experts in musculoskeletal radiology, orthopedic surgery, bone and joint pathology, and rheumatology, reviewed all possible or probable joint safety events identified by the investigator or central reader and all total joint replacements reported during the study and provided the final decision regarding adjudication classification. Joint safety events of rapidly progressive osteoarthritis type 1 or 2 (RPOA1 or 2), subchondral insufficiency fracture, primary osteonecrosis, and pathologic fracture were included in a composite joint safety endpoint. Time-adjusted rates (number of events per 1000 patient-years) of the composite joint safety endpoint and of instances of total joint replacement were compared between the tanezumab and NSAID groups using Poisson models.
