# Supplementary material for: Observed efficacy and clinically important improvements in participants with osteoarthritis treated with subcutaneous tanezumab: results from a 56-week randomized NSAID-controlled study
Source: Arthritis Res Ther. 2022 Mar 29;24:78. doi: 10.1186/s13075-022-02759-0 (PMC8966257; doi:10.1186/s13075-022-02759-0)
Supplement: Supplementary file 2 — Additional file 2: Supplementary Table 1. Pre-study OA analgesic treatment history in randomized participants who received at least 1 SC dose. Table showing analgesic OA treatments used by participants in each treatment group prior to trial enrollment. [file 13075_2022_2759_MOESM2_ESM.docx]

| **Supplementary Table 1.** Pre-study OA analgesic treatment history in randomized  participants who received at least 1 SC dose | | | |
| --- | --- | --- | --- |
| **Analgesics used, n (%) participants** | **Tanezumab 2.5 mg**  ***(N = 1002)*** | **Tanezumab 5 mg**  ***(N =998)*** | **NSAID**  ***(N = 996)*** |
| NSAIDs |  |  |  |
| Ibuprofen | 397 (39.6) | 345 (34.6) | 347 (34.8) |
| Naproxen | 298 (29.7) | 315 (31.6) | 305 (30.6) |
| Celecoxib | 198 (19.8) | 189 (18.9) | 191 (19.2) |
| Meloxicam | 170 (17.0) | 188 (18.8) | 164 (16.5) |
| Diclofenac | 119 (11.9) | 149 (14.9) | 136 (13.7) |
| Naproxen sodium | 96 (9.6) | 106 (10.6) | 106 (10.6) |
| Loxoprofen sodium dihydrate | 34 (3.4) | 26 (2.6) | 31 (3.1) |
| Diclofenac sodium | 29 (2.9) | 31 (3.1) | 26 (2.6) |
| Ketoprofen | 22 (2.2) | 16 (1.6) | 19 (1.9) |
| Acetaminophen/paracetamol |  |  |  |
| Paracetamol | 959 (95.7) | 944 (94.6) | 954 (95.8) |
| Paracetamol; tramadol hydrochloride | 47 (4.7) | 43 (4.3) | 40 (4.0) |
| Tramadol and opioids |  |  |  |
| Tramadol | 375 (37.4) | 363 (36.4) | 405 (40.7) |
| Hydrocodone bitartrate; paracetamol | 125 (12.5) | 140 (14.0) | 113 (11.3) |
| Hydrocodone | 73 (7.3) | 64 (6.4) | 87 (8.7) |
| Oxycodone hydrochloride; paracetamol | 62 (6.2) | 54 (5.4) | 43 (4.3) |
| Codeine phosphate; paracetamol | 44 (4.4) | 46 (4.6) | 51 (5.1) |
| Oxycodone | 36 (3.6) | 37 (3.7) | 37 (3.7) |
| Tramadol hydrochloride | 21 (2.1) | 19 (1.9) | 23 (2.3) |
| Codeine | 15 (1.5) | 21 (2.1) | 16 (1.6) |
| Hyaluronate sodium | 44 (4.4) | 30 (3.0) | 37 (3.7) |
| Table shows treatments received by ≥2% of participants in any treatment group  *NSAIDs* nonsteroidal anti-inflammatory drugs, *OA* osteoarthritis, *SC* subcutaneous | | | |
