# Supplementary material for: Observed efficacy and clinically important improvements in participants with osteoarthritis treated with subcutaneous tanezumab: results from a 56-week randomized NSAID-controlled study
Source: Arthritis Res Ther. 2022 Mar 29;24:78. doi: 10.1186/s13075-022-02759-0 (PMC8966257; doi:10.1186/s13075-022-02759-0)
Supplement: Supplementary file 3 — Additional file 3: Supplementary Table 2. Change in WOMAC Pain, WOMAC Physical Function, and PGA-OA at weeks 56 and 64. Table showing the change from baseline in WOMAC Pain, WOMAC Physical Function, and PGA-OA scores for each treatment group at Weeks 56 and 64 using only observed data. [file 13075_2022_2759_MOESM3_ESM.docx]

| **Supplementary Table 2.** Change in WOMAC Pain, WOMAC Physical Function, and  PGA-OA at Weeks 56 and 64 | | | | |
| --- | --- | --- | --- | --- |
| **Outcome** | **Week** | **Tanezumab 2.5 mg**  ***(N = 1002)*** | **Tanezumab 5 mg**  ***(N = 998)*** | **NSAID**  ***(N = 996)*** |
| WOMAC Pain | 56 | –4.61 (2.10) | –4.50 (2.27) | –4.63 (1.94) |
|  | 64 | –3.47 (2.45) | –3.12 (2.40) | –3.85 (2.07) |
| WOMAC Physical Function | 56 | –4.58 (2.05) | –4.46 (2.26) | –4.63 (1.93) |
|  | 64 | –3.42 (2.40) | –3.12 (2.41) | –3.81 (2.12) |
| PGA-OA | 56 | –1.20 (1.00) | –1.12 (1.03) | –1.26 (0.95) |
|  | 64 | –0.79 (0.96) | –0.64 (0.98) | –0.95 (0.96) |
| Table shows mean (SD) change from baseline, using only observed data (N as follows: tanezumab 2.5 mg = 444 at Week 56 and 437 at Week 64, tanezumab 5 mg = 433 at Week 56 and 419 at Week 64, NSAID = 444 at Week 56 and 445 at Week 64)  *PGA-OA* Patient Global Assessment of Osteoarthritis, *SD* standard deviation, *WOMAC* Western Ontario and McMaster Universities Osteoarthritis Index. | | | | |
