# Supplementary material for: Observed efficacy and clinically important improvements in participants with osteoarthritis treated with subcutaneous tanezumab: results from a 56-week randomized NSAID-controlled study
Source: Arthritis Res Ther. 2022 Mar 29;24:78. doi: 10.1186/s13075-022-02759-0 (PMC8966257; doi:10.1186/s13075-022-02759-0)
Supplement: Supplementary file 4 — Additional file 4: Supplementary Table 3. Amount (mg) of rescue medication use per week. Table showing the amount of rescue medication used in each treatment group at Weeks 2, 4, 8, and 16. [file 13075_2022_2759_MOESM4_ESM.docx]

| **Supplementary Table 3. Amount (mg) of rescue medication use per week^a^** | | | | |
| --- | --- | --- | --- | --- |
| **Week** |  | **Tanezumab**  **2.5 mg**  ***(N = 1002)*** | **Tanezumab**  **5 mg**  ***(N = 998)*** | **NSAID**  ***(N = 996)*** |
| **2** | LS mean (95% CI) | 2880.3 (2265.2, 3662.5) | 2898.7 (2274.4, 3694.4) | 3310.5 (2596.3, 4221.0) |
|  | Versus NSAID |  |  |  |
|  | LS mean ratio (95% CI) | 0.87 (0.66, 1.15) | 0.88 (0.66, 1.16) |  |
|  | *p* value | 0.335 | 0.360 |  |
| **4** | LS mean (95% CI) | 2107.8 (1591.4, 2791.8) | 1946.5 (1472.3, 2573.4) | 2814.1 (2128.0, 3721.5) |
|  | Versus NSAID |  |  |  |
|  | LS mean ratio (95% CI) | 0.75 (0.54, 1.04) | 0.69 (0.50, 0.96) |  |
|  | *p* value | 0.085 | 0.028 |  |
| **8** | LS mean (95% CI) | 1995.6 (1453.8, 2739.3) | 1628.8 (1192.9, 2224.2) | 2839.7 (2087.3, 3863.4) |
|  | Versus NSAID |  |  |  |
|  | LS mean ratio (95% CI) | 0.70 (0.49, 1.01) | 0.57 (0.40, 0.83) |  |
|  | *p* value | 0.060 | 0.003 |  |
| **16** | LS mean (95% CI) | 1696.4 (1188.7, 2420.8) | 1581.6 (1112.2, 2249.1) | 2320.0 (1636.5, 3289.0) |
|  | Versus NSAID |  |  |  |
|  | LS mean ratio (95% CI) | 0.73 (0.48, 1.11) | 0.68 (0.45, 1.03) |  |
|  | *p* value | 0.139 | 0.071 |  |
| ^a^Total dose of acetaminophen (mg) for each week. Data were not assessed after Week 16  *CI* confidence interval, *LS* least squares, *NSAID* nonsteroidal anti-inflammatory drug | | | | |
