# Supplementary material for: Observed efficacy and clinically important improvements in participants with osteoarthritis treated with subcutaneous tanezumab: results from a 56-week randomized NSAID-controlled study
Source: Arthritis Res Ther. 2022 Mar 29;24:78. doi: 10.1186/s13075-022-02759-0 (PMC8966257; doi:10.1186/s13075-022-02759-0)
Supplement: Supplementary file 5 — Additional file 5: Supplementary Table 4. Patient-reported treatment preference for study medication using mPRTI. Table summarizing treatment preference scores in each treatment group at Weeks 16 and 56. [file 13075_2022_2759_MOESM5_ESM.docx]

| **Supplementary Table 4. Patient-reported treatment preference for study medication using mPRTI** | | | | | |
| --- | --- | --- | --- | --- | --- |
| **Question** | **Week** | **Response** | **Tanezumab 2.5 mg**  ***(N = 1002)*** | **Tanezumab 5 mg  *(N = 998)*** | **NSAID**  ***(N = 996)*** |
| Overall, do you prefer the drug that you received in this study to previous treatment?^a^ | 16 | Yes, I definitely prefer the drug that I am receiving now. | 577 (61.4) | 597 (62.6) | 531 (56.7) |
|  |  | I have a slight preference for the drug that I am receiving now. | 141 (15.0) | 169 (17.7) | 158 (16.9) |
|  |  | *p* value versus NSAID | 0.082 | 0.005 |  |
|  | 56 | Yes, I definitely prefer the drug that I am receiving now. | 342 (68.7) | 323 (66.3) | 302 (61.8) |
|  |  | I have a slight preference for the drug that I am receiving now. | 70 (14.1) | 75 (15.4) | 89 (18.2) |
|  |  | *p* value versus NSAID | 0.072 | 0.195 |  |
| Willing to use the same drug that you have received in this study for your osteoarthritis pain?^b^ | 16 | Yes, I would definitely want to use the same drug again. | 627 (66.8) | 641 (67.2) | 560 (59.8) |
|  |  | I might want to use the same drug again. | 138 (14.7) | 154 (16.1) | 169 (18.1) |
|  |  | *p* value versus NSAID | 0.023 | 0.003 |  |
|  | 56 | Yes, I would definitely want to use the same drug again. | 352 (70.7) | 341 (70.0) | 310 (63.4) |
|  |  | I might want to use the same drug again. | 78 (15.7) | 75 (15.4) | 97 (19.8) |
|  |  | *p* value versus NSAID | 0.027 | 0.184 |  |
| ^a^Responses were scored on 5-point Likert scale from 1 = “No, I definitely prefer my previous treatment” to 5 = “Yes, I definitely prefer the drug that I am receiving now.” For simplicity, only responses 4 (slight preference) and 5 (definitely prefer) are shown in the table  ^b^Responses were scored on 5-point Likert scale from 1 = “No, I definitely would not want to use the same drug again” to 5 = “Yes, I would definitely want to use the same drug again.” For simplicity, only responses 4 (might want to) and 5 (definitely want to) are shown.  Each row shows the number (% of participants) with that particular response; with percentages based on the number of participants with available data at week 16 or week 56  *mPRTI* Patient-Reported Treatment Impact Assessment-Modified; *NSAID* nonsteroidal anti-inflammatory drug | | | | | |
