# Supplementary material for: Observed efficacy and clinically important improvements in participants with osteoarthritis treated with subcutaneous tanezumab: results from a 56-week randomized NSAID-controlled study
Source: Arthritis Res Ther. 2022 Mar 29;24:78. doi: 10.1186/s13075-022-02759-0 (PMC8966257; doi:10.1186/s13075-022-02759-0)
Supplement: Supplementary file 6 — Additional file 6: Supplementary Table 5. Global treatment satisfaction with study medication using the TSQM. Table summarizing treatment satisfaction scores in each treatment group at Weeks 16 and 56. [file 13075_2022_2759_MOESM6_ESM.docx]

| **Supplementary Table 5.** Global treatment satisfaction with study medication using the TSQM | | | | |
| --- | --- | --- | --- | --- |
| **Week** |  | **Tanezumab 2.5 mg**  ***(N = 1002)*** | **Tanezumab 5 mg  *(N = 998)*** | **NSAID**  ***(N = 996)*** |
| **16** | n | 939 | 954 | 936 |
|  | Mean (SD) | 72.0 (22.9) | 72.3 (23.2) | 68.9 (23.2) |
|  | Median | 75.0 | 75.0 | 66.7 |
|  | LS mean (SE)  [95% CI] | 70.3 (1.0)  [68.4, 72.3] | 70.7 (1.0)  [68.8, 72.6] | 67.1 (1.0)  [65.2, 69.1] |
|  | Versus NSAID |  |  |  |
|  | LS mean difference (SE)  [95% CI] | 3.2 (1.1)  [1.1, 5.3] | 3.6 (1.0)  [1.5, 5.6] |  |
|  | *p* value | 0.003 | 0.001 |  |
| **56** | n | 498 | 487 | 489 |
|  | Mean (SD) | 76.3 (20.8) | 73.7 (22.3) | 74.1 (21.0) |
|  | Median | 83.3 | 75.0 | 75.0 |
|  | LS mean (SE)  [95% CI] | 75.3 (1.3)  [72.8, 77.8] | 73.4 (1.3)  [70.9, 75.8] | 73.4 (1.3)  [70.9, 75.9] |
|  | Versus NSAID  LS mean difference (SE)  [95% CI] | 1.9 (1.3)  [-0.6, 4.5] | 0.0 (1.3)  [-2.6, 2.6] |  |
|  | *p* value | 0.137 | 0.996 |  |
| Global treatment satisfaction score ranges from 0 to 100, with 100 being the best (most satisfied) score  *CI* confidence interval, *LS* least squares, *NSAID* nonsteroidal anti-inflammatory drug, *SD* standard deviation, *SE* standard error, *TSQM* Treatment Satisfaction Questionnaire for Medication v.II | | | | |
