# Supplementary material for: Observed efficacy and clinically important improvements in participants with osteoarthritis treated with subcutaneous tanezumab: results from a 56-week randomized NSAID-controlled study
Source: Arthritis Res Ther. 2022 Mar 29;24:78. doi: 10.1186/s13075-022-02759-0 (PMC8966257; doi:10.1186/s13075-022-02759-0)
Supplement: Supplementary file 7 — Additional file 7: Supplementary Table 6. Serious musculoskeletal and connective tissue AEs during the 56-week treatment period. Table summarizing serious musculoskeletal and connective tissue AEs in each treatment group during the 56-week treatment period. [file 13075_2022_2759_MOESM7_ESM.docx]

| **Supplementary Table 6.** Serious musculoskeletal and connective tissue AEs during the 56-week treatment period | | | |
| --- | --- | --- | --- |
| **N (%) participants** | **Tanezumab 2.5 mg**  ***(N = 1002)*** | **Tanezumab 5 mg**  ***(N = 998)*** | **NSAID**  ***(N = 996)*** |
| Any event  Arthralgia  Arthritis  Back pain  Intervertebral disc protrusion  Lumbar spinal stenosis  Musculoskeletal pain  Osteoarthritis  Osteonecrosis  Pain in extremity  Rapidly progressive osteoarthritis  Rotator cuff syndrome  Spinal osteoarthritis  Spondylolisthesis  Subchondral insufficiency fracture  Thoracic spinal stenosis | 18 (1.8)  4 (0.4)  0  0  0  1 (0.1)  0  9 (0.9)  0  1 (0.1)  3 (0.3)  0  0  0  1 (0.1)  0 | 45 (4.5)  9 (0.9)  1 (0.1)  1 (0.1)  2 (0.2)  1 (0.1)  1 (0.1)  17 (1.7)  2 (0.2)  0  11 (1.1)  0  0  1 (0.1)  4 (0.4)  1 (0.1) | 10 (1.0)  0  0  1 (0.1)  1 (0.1)  1 (0.1)  1 (0.1)  4 (0.4)  0  0  0  1 (0.1)  1 (0.1)  0  2 (0.2)  0 |
| *AE* adverse event, *NSAID* nonsteroidal anti-inflammatory drug | | | |
